# The Norwegian National Summary Care Record: a qualitative analysis of doctors’ use of and trust in shared patient information

**DOI:** 10.1186/s12913-018-3069-y

**Published:** 2018-04-06

**Authors:** Kari Dyb, Line Lundvoll Warth

**Affiliations:** Norwegian Centre for E-health Research, Siva Innovation Centre Breivika, Sykehusveien. 23, 9019 Tromsø, Norway

**Keywords:** Summary care record, Doctors use and experiences, Implementation, Information sharing, Trust, Qualitative analysis

## Abstract

**Background:**

This paper explores Norwegian doctors’ use of and experiences with a national tool for sharing core patient health information. The summary care record (SCR; the *Kjernejournal* in Norwegian) is the first national system for sharing patient information among the various levels and institutions of health care throughout the country. The health authorities have invested heavily in the development, implementation and deployment of this tool, and as of 2017 all Norwegian citizens have a personalised SCR. However, as there remains limited knowledge about health professionals’ use of, experiences with and opinions regarding this new tool, the purpose of this study was to explore doctors’ direct SCR experiences.

**Methods:**

We conducted 25 in-depth interviews with 10 doctors from an emergency ward, 5 doctors from an emergency clinic and 10 doctors from 5 general practitioner offices. We then transcribed, thematically coded and analysed the interviews utilising a grounded theory approach.

**Results:**

The SCRs contain several features for providing core patient information that is particularly relevant in acute or emergency situations; nonetheless, we found that the doctors generally used only one of the tool’s six functions, namely, the pharmaceutical summary. In addition, they primarily used this summary for a few subgroups of patients, including in the emergency ward for unconscious patients, for elderly patients with multiple prescriptions and for patients with substance abuse conditions. The primary difference of the pharmaceutical summary compared with the other functions of the tool is that patient information is automatically updated from a national pharmaceutical server, while other clinically relevant functions, like the critical information category, require manual updates by the health professionals themselves, thereby potentially causing variations in the accuracy, completeness and trustworthiness of the data.

**Conclusion:**

Therefore, we can assume that the popularity of the pharmaceutical summary among doctors is based on their preference to place their trust in – and therefore more often utilise – automatically updated information. In addition, the doctors’ lack of trust in manually updated information might have severe implications for the future success of the SCR and for similar digital tools for sharing patient information.

**Electronic supplementary material:**

The online version of this article (10.1186/s12913-018-3069-y) contains supplementary material, which is available to authorized users.

## Background

In recent years, shared electronic health records have begun to be introduced in a variety of countries for the exchange of health care–related data, including medication, allergies, medical histories, laboratory reports, referral letters and discharge summaries [1]. The Norwegian summary care record (SCR), known as the *Kjernejournal* in Norwegian, is a new, national, digital tool that gives all health professionals access to selected health information regardless of where a patient is treated. This is the first national, digital tool for sharing patient information across all institutions and levels of care in Norway [2, 3]. The purpose of the SCR is to increase patient safety through the quick and secure access of health care professionals to structured, core information about each patient. As of the end of 2017, all Norwegian citizens have a personalised SCR. However, despite the huge financial investment and resources devoted to its development, implementation and deployment, the SCR is still not a routinely used tool in the Norwegian health care sector.

However, challenges with implementing programmes such as the SCR is not exclusive to the Norwegian context. Implementing organisational change in health care systems is complex, difficult to manage and liable to generate unintended consequences [4, 5]; hence, a limited or lack of use of new digitised tools like the SCR is nothing new [6]. Internationally, several recent studies on information technology investments in the health care sector show that despite huge governmental and corporate investments and promises to deliver quicker, cheaper and safer patient services, there has been a litany of delays, compromises and failures [7–9]. The track record for technology programmes continues to be poor because of the combined problems of a lack of adoption and abandonment by individuals and difficulties with scale-up and spread [10]. The literature illustrates that it has been particularly difficult to implement large-scale interventions [5, 11] and national digital tools [7, 10]. Large-scale programmes in particular are plagued by a range of interrelated factors – including those that are technical, social, organisational and socio-political – that require complex strategic planning incidental with systemic organisational change [4, 10–13].

The large-scale implementation research on doctors’ behaviours and attitudes towards digital systems for data sharing is particularly relevant for our study. However, there is limited research on SCRs, and much of this research is centred on electronic health records (EHRs). The EHR research illustrates, for example, that the implementation of large-scale EHR programmes sometimes fails due to a lack of integration into practices and organisations [14], and the existing studies point to doctors’ attitudes as a critical factor for EHR implementation since doctors can choose to simply not access them [15]. Whether doctors accept or reject EHR implementation depends on their acceptance of their work practices being transformed, and the likelihood of acceptance will be increased if implementers address their concerns [16]. Other common barriers for doctors’ EHR adoption are lack of time, absence of computers skills and concerns that vendors are not qualified to provide proper servicing of the technology over time [17].

In regard to the SCR, most of the research originated in the United Kingdom and focused on evaluating implementation programmes. Some of the studies highlight the challenges and complexities surrounding the implementation and use of such a tool, emphasising that the SCR is not a simple plug-in technology and that health personnel might choose to not use it [18–20]. There is no direct evidence of improved patient safety, although some findings are consistent with a rare but important positive impact on preventing medication errors [20]. However, another study found that SCRs increase patient safety, improve the quality and effectiveness of care and save health care staff and their organisations time and money [21]. In Scotland, an evaluation of the impact of the “Key Information Summary” on general practitioners and out-of-hours clinicians reported benefits for specific groups of patients and for the out-of-hours clinicians [22].

In the Norwegian SCR context, there are two other papers that have focused on the SCR. In the first paper, the authors studied the piloting of the SCR and conclude that widespread electronic communication and collaboration require a flexible documentation practice that can be adjusted to meet the needs of a wide range of actors [23]. The second paper focused on the implementation phase of the technology and show that while project leaders considered their work to be finished after the technology implementation, the doctors viewed the SCR as one of several small steps towards a large, still-to-come national tool for shared patient information [24]. A third Norwegian paper is also relevant to the SCR context in that it focused on information access and information needs in inpatient emergency departments and illustrated that Norwegian health professionals have a clear clinical need for selected, up-to-date, easily-accessible patient summaries [25].

To date, there remains a limited understanding of how health professionals in Norway use and experience the SCR even though this knowledge is in great demand. Therefore, inspired by the grounded theory approach, the objective of this study was to explore doctors’ use of and experiences with the SCR. Our aim is to contribute to the existing empirical knowledge on doctors’ direct SCR experiences in Norway, as well as to enrich the general debate on top-down governmental implementations of large-scale programmes. The research question of this study is as follows: How do doctors use and experience the SCR, and what does the new tool represent for them?

### Norwegian digital health information and the SCR

Norwegian health and social care services are based on the classical Scandinavian welfare model, which combines financing and the provision of universally accessible services mainly within the public sector. It is organised into primary health care and long-term care on the one hand and in-hospital care and specialist services on the other [26]. General practitioner offices and the municipal day and night emergency clinics are part of the primary health and care sector, while hospitals fall under specialist health and care services.

In relation to doctors’ use of and experiences with digital health information, Norwegian general practitioners were among the first in the world to implement EHRs, and today most general practitioners’ offices have their own EHR systems [27]. They can access the SCRs of their patients by clicking on an icon that is included in their local EHR system. Currently, in the primary health and care sector, the tool is accessible for general practitioners and for doctors and nurses in emergency clinics. However, the health authorities plan to expand the tool to the municipal nursing services by 2018. Compared to general practitioners, hospitals have had a slower pace of EHR implementation, but all Norwegian hospitals now have EHR systems [27]. Just like the primary health and care sector, the SCR appears as an icon in a hospital’s EHR system, and the tool is accessible for all doctors and nurses on staff. The SCR icon is identical regardless of the EHR system. A blue icon signals that the SCR does not contain any registered critical information about the patient, while a red icon signals that doctors have registered critical information like allergies, implants or relevant chronic conditions in the SCR. The purpose of a red icon is to alert health care professionals that a patient has health issues that they should be aware of, particularly in emergencies or in situations when a patient or next of kin cannot provide this information. The objective of the tool is to provide easy access to information that is useful in everyday practice and potentially life-saving in emergencies [2].

In order to access the SCR, doctors and nurses need authorisation from the health authorities, which they gain by taking an online test. Health professionals do not need consent from a patient to access their SCR, although all SCR activity is logged and traceable. Only doctors and nurses with a legitimate need for core information about a patient have the legal right to access that patient’s SCR. Patients can access their SCR any time through a government patient platform called Helsenorge.no.

It is important to note that the SCR does not replace the local EHR systems in primary or in secondary care settings. The new national tool is designed to serve as a supplementary information system, providing core information for all Norwegian citizens regardless of where they receive treatment. A patient’s SCR contains the following: 1) personal data, like a patient’s address, next of kin and their general practitioner’s name and contact information; 2) a pharmaceutical summary; 3) potential critical information, such as allergies, implants and relevant chronic conditions if registered by a doctor; 4) admission history from Norwegian hospitals; and 5) information that the patient has registered himself or herself. Table 1 illustrates the main functions of the SCR and its content.

**Table 1:** The Six Main Functions of the Summary Care Record

Summary	SCR	Provides health personnel with a quick summary of critical information about the patient, such as any medications, diagnosed illnesses and illnesses registered by the patient
About the patient	Population register/national GP register	Provides personal information, such as the patient’s address, material status, family members and GP
Pharmaceutical summary	Prescription provider	Provides prescribed pharmaceutical information collected from pharmacies and valid e-prescriptions; includes prescribed consumer goods
Critical information	Attending physician	Provides information about serious allergies, special disorders and other important information in a structured form
Patient history from the specialist health care	Norwegian patient registrar	Provides the time and place of hospitalisations and visits to specialists
The patient’s own registration	Patient	Provides information on relatives and other contacts, special communication needs and diseases

## Method

In this paper, we have limited our focus to how doctors use and experience the SCR. To gather data on their opinions of the new tool, we undertook a qualitative study using in-depth interviews as the methodological approach [28–32]. We did the fieldwork and data collection in 2016, from September 4 to 10, in the Trondheim municipality; the city of Trondheim has the longest experience with the national SCR, as this is where it was initially introduced in 2013. The doctors were all experienced health professionals with long-term knowledge and familiarity with the SCR, although not all of them had used the tool since it was introduced in 2013. The doctors’ responses were based on their personal experiences and opinions.

### In-depth interviews with the doctors

Ten regular general practitioners from 5 different medical offices in Trondheim, 5 doctors from the emergency clinic in the Trondheim municipality and 10 doctors from the emergency ward at St. Olav’s Hospital were interviewed. The informants were selected with the goal of gathering knowledge on doctors’ use of and experiences with the new tool in both primary and specialist service settings.

A research assistant recruited the informants. The assistant phoned all 37 regular general practitioner offices in Trondheim, described the study and asked for an interview. We received positive responses from 5 different medical offices, and we ultimately interviewed 10 general practitioners (2 women and 8 men).

At the municipal emergency clinic and hospital emergency ward, the research assistant phoned the directors of the two units and asked for help with providing information to the staff about the study and recruiting doctors who were on duty during the specific week that was committed to data collection. The research assistant then sent e-mails to all the doctors on duty during that week, describing the study and asking for their participation in qualitative interviews about the SCR. A total of 15 doctors (1 woman and 14 men) responded quickly, agreeing to be interviewed.

A sociologist and a medical doctor, both trained qualitative researchers, conducted the interviews. The sociologist, who is the first author of this paper, had the main responsibility for the research process, while the medical doctor played an important role as an insider in the field of medicine. His contributions included legitimatising the research project with the doctors and translating the medical terminology and practices. All interviews were done at the workplaces of the informants in either the doctor’s office, a meeting room or a staff break room. The 10 general practitioners received reimbursement for their time (equivalent to the standard wage scale for patient consultations). We audiotaped and then transcribed all of the interviews. The data material consisted of 25 transcribed interviews of 20 to 60 min each in duration.

At the start of the interviews, we introduced the overall and main question for the study, “What does the SCR mean to you?”, and emphasised that there were no right or wrong answers. Since we were interested in the doctors’ personal stories of the SCR, we encouraged them to reflect on the question and talk freely about their opinions. We also said that we were not going to interrupt but would take notes for follow-up questions [33]. Open questions encourage informants to talk about what they perceive as relevant and what information they want to convey in the manner and order of their choosing. This type of interview helps a researcher obtain answers to questions that he/she has not thought of asking [34, 35], and it involves a shift for the interviewer from being privileged and knowledgeable to being passive and reflective [29]. We asked follow-up questions when we felt this was necessary. The English version of the interview guide is saved as Additional file 1.

Since our methodology was motivated by what can be labelled as a minimalist passive interviewing approach [33], which was inspired by Glaser and Straus’ grounded theory [36], our data analysis was based on analytic induction. The first author read all the transcripts and noted possible empirical and analytical themes; these themes were then discussed among the research group consisting of the two authors and the medical doctor who participated in the interviews. Based on a few particularly rich transcripts we then developed a coding framework, which we continuously discussed, tested and modified to check if it could be applied to all the transcripts. The doctors’ stories about the SCR were surprisingly homogeneous, and the results we describe here represent some aspects that constitute a pattern in the doctors’ experiences, use of the SCR and what the SCR represented for them. The authors have translated the included quotes from Norwegian to English.

We applied for approval from the Regional Committee for Medical and Health Research Ethics, but it was not required for this study. The data protection officer at the University Hospital of Northern Norway did approve the study, and all the doctors who participated as informants signed an informed consent form.

## Results

In this section, we present our findings regarding the main question that we asked the informants, “What does the SCR mean to you?,” the purpose of which was to explore how the doctors used and experienced the SCR and what the new tool represented to them.

### A blue SCR icon does not necessarily mean that the patient does not have critical information

As we described in the background section, an SCR is accessible from the doctor’s local EHR system and appears as either a blue or a red icon. A red icon is as an alert, signalling that the SCR holds critical information about a patient. Most of the doctors in our study said that they usually glanced at the SCR icon to check its colour, and if the icon was red, which was extremely rare, they knew the SCR contained critical information about the patient. In such situations, the doctors working in the emergency clinic and at the emergency ward opened the SCR to check for critical information (e.g., informants 2, 3 and 5). On the other hand, some of the regular general practitioners (e.g., informants 20 and 23) said that a red icon usually mirrored critical information the doctor had already registered herself/himself. Each of the general practitioners said they had one or two patients to whom they had entered critical information. None of the doctors had experienced that the alert had helped save a patient life.

If the SCR icon was blue, which was by far the most common situation, they felt that the tool did not provide much information. All the doctors emphasised that a blue SCR icon did not necessarily mean that the patient did not have critical information; it just meant that no one had entered such information into the SCR. Consequently, the doctors did not think that the critical information function had been updated and was correct.

### The SCR represents access to the pharmaceutical summary

Of the six functions in the SCR, the doctors emphasised the pharmaceutical summary. The frequency of use of this function varied, but the doctors mainly described the SCR as a tool for gaining insight regarding registered prescriptions and which pharmaceutical medications the pharmacies had supplied to patients. One doctor in the emergency ward described the SCR as one of several sources they use to get an overall picture of medication:

Yes, that is something I use. Yes, it is. I probably use it mostly, mostly in relation to pharmaceuticals and so on. That is what I mostly look at. And so it would be one of several sources where you try to get an overall impression of what is correct and up to date. (Informant 9, emergency ward)

When exploring what the SCR represented for the doctors in the emergency ward, the emergency clinic and 5 general practitioner’s offices in Trondheim, almost all (e.g., Informants 9, 7 and 12) emphasised the pharmaceutical summary. They all highlighted the value of seeing the medication that had been prescribed and the prescriptions that were registered for a patient. Several doctors stressed medication errors as a tremendous threat to patients’ safety. One doctor (Informant 5) talked about medication error as one of the largest threats to patients’ safety, and most of the doctors welcomed the new tool for this exact reason; that is, preventing medical errors. However, the same doctors also emphasised that the pharmaceutical summary in the SCR did not provide a complete list of a patient’s medication and that they did not put complete trust in the new tool. One doctor put it this way:

At the same time, the SCR do not provide a solid medication list. It indicates what you can assume the patients is taking based on prescriptions from the GP and hospitals. That is the nature of the information in the SCR. (Informant 5, emergency ward)

### The pharmaceutical summary is most useful in the emergency ward

Although the doctors in the emergency ward, emergency clinic and general practitioner offices all described the pharmaceutical summary as being useful, they also emphasised that it was most useful in the emergency ward (21 out of 25 doctors). The following quotes illustrate this.

I mainly use it when I am in the emergency ward and just occasionally when I am on regular duty. We mostly use it in connection with medication and medication lists. We check prescriptions and supplies, and so on, to get a better overview of what medicine people actually have at home and which medication they have been given – what is written on the package. (Informant 4, emergency ward)

This statement, made by a doctor working in the hospital who used the tool occasionally in everyday practice, stresses that it is the most useful when working in the emergency ward. It illustrates how the doctor used the SCR to get a picture of the medications prescribed to a patient and any supplies a patient had picked up from the pharmacies. A fellow doctor working in the emergency clinic (Informant 3) shared the perception of the pharmaceutical summary as most useful in the emergency ward:

It is sort of an emergency tool, and it is supposed to be that – of the patients, we receive here (the emergency clinic), how many are actually very acute? What is more, there should not be many in an emergency clinic, either; otherwise, we have used bad triage on the way in. If they are very ill, they should be in the emergency ward. (Informant 3, emergency clinic)

The perception of the pharmaceutical summary as an emergency tool was common among the general practitioners as well. Several general practitioner said they found it difficult to see the personal value of the SCR and thought the SCR was most useful in emergency wards (e.g., Informants 6 and 21). One general practitioner put it this way:

We [our medical centre] were asked to pilot it [the SCR], to enter critical information and to use it. I was at the disposal of the health authorities, so to speak, as a regular general practitioner – to explore the tool’s value for me. And, it is less and less, almost zero. I enter information, but I do not look up anything [in the SCR]. The SCR works as a tool for hospitals to look into what medication we [GPs] prescribe. That is how the tool is used. No allergies or rare diseases, etc., it is nothing, but the use of the pharmaceutical summary is useful for some. (Informant 16, regular general practitioner)

Here, we see that the doctors, regardless of where they worked, emphasised that the SCR is most useful for health professionals working in hospitals; that is to say, as a pharmaceutical summary. In addition, the last statement also illustrates how the critical information function has limited value for doctors, particularly for general practitioners. According to one doctor (Informant 16), general practitioners enter critical information into the SCR but do not use the tool for checking allergies or rare diseases. This regular general practitioner had never seen critical information registered by a hospital doctor, and, hence, the critical information function had limited value, almost zero. This doctor’s experience is in line with the general findings from our study: While most of the general practitioners said they went through their patient lists and consistently updated their SCRs with critical information, the hospital doctors, as we will exemplify later, did not claim do so.

### The pharmaceutical summary is particularly useful for three groups of patients

When the doctors mentioned the pharmaceutical summary as being useful, they emphasised its usefulness for three patient groups: 1) unconscious patients, particularly those with no hospital records, as that meant that they had no information for them on file; 2) elderly patients who used multiple pharmaceutical products and had trouble giving a clear account of their medications; and 3) patients with a history of substance abuse. For the first group, the pharmaceutical summary can provide important information in situation where a patient or his/her next of kin is not able to provide it, such as a stroke patient:

Yes, the SCR is good in that I use it primarily when there are unconscious patients you do not know anything about … A classic example is a thrombolysis alarm for stroke. You know nothing about the patient who is on vacation from Frøya [Island] and arrives here and is unable to talk or has aphasia or dysarthria. You wonder whether this person is taking Marevan or other blood-thinning medications that will have major consequences for treatment. And in those cases, it is simply a matter of looking up the medication, that is, the medication supplied by the pharmacies. (Informant 19, emergency ward)

While this doctor described how she/he use the tool to get an overview of pharmaceuticals for unconscious patients, another doctor emphasised the tool’s usefulness for elderly patients with complex medications:

In my opinion, the summary record is a useful tool, especially in connection with medication. I actually only use it for that … [for] the elderly who use many different types of medicine, when the lists are partly different, and when it is uncertain whether the patient has received such and such a pharmaceutical product. (Informant 18, emergency ward)

In addition to unconscious patients with no hospital records and elderly patients who used multiple pharmaceutical products, the general practitioners and the doctors in the emergency clinics and wards all described the SCR’s utility in relation to drug abuse. One doctor at the emergency clinic highlighted its usefulness for preventing multiple prescriptions:

It is very good that you can see [for] those who come to get medicine that they may not need so much and [those] who want tranquilisers or painkillers, and then it turns out they got that a few days ago from their regular general practitioner. That is the best thing about it. If there is one thing that is most useful, then it is precisely that. (Informant 7, emergency clinic)

Both the doctors at the emergency clinic and the doctors working in the emergency wards found the pharmaceutical summary particularly useful for the treatment of patients with a history of drug abuse. One said:

The summary care record can make it safer. At least we can confirm what they have got and if the patient is telling the truth; for example, patients with a history of substance abuse who might claim they get 20 paralgin forte from their GP. We can enter the SCR and see that this is not correct and that the situation is 2 per day. You get an indication of what is correct, but you cannot trust it 100%. (Informant 5, emergency ward)

The common benefit for the doctors treating patients with multiple, complex prescriptions and patients not capable or willing to communicate reliably was access to the patient’s current medications and prescriptions through the pharmaceutical summary in the SCR.

### The SCR represents additional work

According to the interviews, the hospital-based doctors felt that there were severe deficiencies with the existing systems for information exchange. Much more than the general practitioners, these doctors described an everyday struggle with gaining access to correct and updated information, including patients’ medications, allergies, relevant chronic conditions and medical histories. Limited access to information was common in the hospital settings, and several hospital doctors (e.g., Informants 4 and 9) claimed that a shared EHR system for primary and secondary care might mitigate such deficits. However, the same doctors stressed that it was not sufficient to just implement a new computer system (e.g., the SCR) as the doctors were already bored of new, competing information systems and felt that the system did not offer a complete service and that it just added data-entry and record-keeping duties to their already busy schedules. One doctor said:

We are getting more and more computer systems. To implement new, digitised information systems seems trendy in the health and care sector. I think that people (health personnel) find it frustrating with all the overlapping systems, as you have to make duplicates [of patient records]; e.g., an implant. You must enter it in the hospital EHR. In addition, most implants have their own quality record system to which you have to report it, you must also record it as critical info in Doculife and you should record it in the SCR. This means you can record the same implant five times, and that is not doable in everyday clinical practice – you do not have the time! (Informant 5, emergency ward)

When the systems overlapped, doctors had to prioritise between them, and they usually chose to record data in the same systems used by their closest colleagues. The same doctor continued:

You record it in the systems that you feel is useful, the ones you use yourself and the systems that your closest colleagues use, and you drop the parallel systems, which are a double workload and repetitive work. The existing computer systems requires a double workload, when it theoretically should be possible to simplify the work … You cannot just establish a parallel system; that is what they (the health authorities) do – they continuously produce new systems. (Informant 5, emergency ward)

Here, a hospital doctor, immediately after expressing a need for shared systems and easier access to information, emphasised how the continuous implementation of new technologies like the SCR did not automatically lead to improvements but rather created an additional workload and a need to prioritise among the various systems.

## Discussion

The results show that the doctors in our study routinely glanced at their patients’ SCR icons to check the colour but put limited trust in the data quality of this function. They also illustrate that the doctors preferred the pharmaceutical summary to the other functions, even if it did not provide a complete medication list and they mainly used it in the emergency ward and for specific groups of patients. That the Norwegian SCR is particularly beneficial for specific groups of patients is in line with the evaluation of the impact of the “Key Information Summary” in Scotland [22]. Challenges with the large-scale implementation of programmes [5, 11] and health care personnel, particularly doctors, not always using digital tools for information sharing as planned are established knowledge [1, 37]. The implementation literature has reported on numerous frameworks [5, 10, 38] for assisting governments and corporations with new interventions and technology in the health care sector for exactly these reasons. However, our study also offers new insight into how doctors differentiate among the functions in the same tool – while they use some of the functions, others are less trusted. Trustworthiness is a particularly important issue in relation to doctors’ use of and experiences with the SCR in Norway.

A plausible reason for why the doctors were less interested in the remaining functions of the SCR (e.g., about the patient, critical information, the patient’s history of specialist health care and the patient’s personal registration) is that information of an administrative nature – like the patient’s address, phone number and family members – in the function, about the patient, is more relevant for nurses or administrative personnel than for doctors, at least in hospital settings. The same argument can be applied to the patient’s registration function, which can contain information about relatives and other contacts, special communication needs, diseases and preferences. Therefore, nurses’ use of and experiences with the SCR is a potential focus of future studies. This is particularly relevant since the health authorities’ implementation and deployment plan is to open the SCR to municipal nursing services in primary care by the end of 2018 and studies have found that nurses tend to not adopt technology-enabled “data sharing” [39].

Information about serious allergies, special disorders, implants and other important core information is listed under the critical information function, which was categorised as a distinct function in the SCR by policy makers. In fact, the rationale behind the government’s proposal for the SCR was the following: “For some patients it is of uttermost importance for health personnel to get access to information about ongoing treatment and relevant clinical history. In such situations, today’s existing system for information sharing is not adequate” [3]. The SCR icon is even designed to change colour from blue to red to alert health professionals if it the SCR contains critical information about a patient. We can argue that since the doctors routinely checked the colouring of the SCR icon, they were using the tool as planned prior to implementation. However, our study demonstrates that 3 years after the initial implementation, the doctors still did not trust the colouring system of the SCR. To them, a blue icon did not equal a lack of critical information.

This mismatch between the political and policy focus on the need for both pharmaceutical and critical clinical information and our findings that the doctors in this study exclusively trusted the pharmaceutical summary is interesting on several levels. It illustrates how new technologies do not automatically lead to improvements in accompanying work practices, organisational structures or models of care [9] even when there is a known clinical need for the information provided by the new tool. The results also illustrate that a new electronic record system is not merely a container for information but instead accumulates and transforms work [40]. For example, for the doctors in our study, the critical information function represented additional work. Parallel information systems and easy access to information can lead to what MacNeill and colleagues (2014) described as a ‘tsunami’ of patient data [41].

As stated previously, to keep the critical information function updated, doctors must manually enter information about serious allergies, special disorders and other important information on an on-going basis. This means that the national SCR depends on all Norwegian doctors’ collective willingness to consistently enter relevant information. Our study indicates that while many general practitioners went through their patient lists and consistently updated the SCR with critical information, many hospital doctors did not do this. In hospitals, emergency wards in particular, doctors treat many unfamiliar patients. This is further complicated by their limited free time, increased documentation demands and several overlapping recording and information systems. We therefore argue that if a doctor does not record data in the SRC, then they will not think that other doctors are doing so, either. Hence, they will not trust the quality of the manually updated data in the SCR.

It is our interpretation that the doctors trust, or at least highly regard, the automatically generated information in the pharmaceutical summary. However, the lack of commitment of the doctors, particularly those working in hospitals, to consistently update the critical information in the SCR has resulted in unreliable data and a lack of trust in this function of the new tool. Trust is one of the central features in the health care sector, particularly in the context of patient-physician relationships. There is a long list of studies that have focused on issues related to how rapid changes in health care systems, such as digitalisation, are feared by many and threaten patients’ trust in their physicians [42]. In relation to information systems, trust was not included as a central factor in a recent (2017) systematic review [43] of user acceptance factors of hospital information systems. When it comes to shared health information, legal issues concerning privacy, security and liability are heavily debated in the literature [44, 45]. Which data sources they prefer or trust the most is a much-less-explored topic. Our study indicates that when designing new tools for sharing data to improve patient safety, which data sources health personnel find trustworthy need to be further explored.

## Limitations

We conducted the study in the city of Trondheim in September 2016, which ensured that we could access experience-based knowledge from doctors working in a city with more than 3 years of familiarity with the tool. To understand technologies with regard to practical experience and utilisation, one must first understand the places in which they are used or not used [46]. This knowledge raises questions about the significance of choosing Trondheim as our site of research and if our analyses are applicable for other towns, health regions and countries. For such questions, we need similar in-depth studies of doctors’ direct SCR experiences in other towns, health regions and countries.

The health authorities implemented the SCR in Trondheim in 2013, while we interviewed 25 doctors’, in the same city in 2016. While time is of the essence when it comes to assessing health personals behaviour and attitudes towards new interventions in health care and determining if they become normalised [38], it may seem that studying doctors’ experiences with a national intervention after only 3 years is too soon. However, we believe that a qualitative analysis of 25 doctors’ trust, or lack of trust, in manually updated data sources they do not record information in themselves is relevant as a qualitative phenomenon. In particular, the relevance of trust in relation to data quality and shared information is of national and international importance.

## Conclusion

Compared to the political and policy impetus for the SCR [1, 2], which points to a need for a system that gives health professionals fast access to core information across all levels and organisations in the Norwegian health care sector, this study shows that the expected benefits of the SCR are still primarily untapped. Our study illustrates that even if doctors checked the colouring of the SCR icon regularly, they placed limited trust in the quality of the critical information function. It also illustrates that the doctors preferred the pharmaceutical summary to the other functions even if it did not provide complete medication lists for their patients. As a to-the-point formulation, many doctors equalised the SCR with the pharmaceutical summary, which was a functional tool for most of them, but particularly for those working in hospitals, such as in the emergency ward and when treating specific groups of patients.

Our findings also show that the doctors’ preference for the pharmaceutical summary is not solely related to the kind of information they need in clinical practice. Surely, doctors’ limited use of the functions of an administrative nature might be related to work practices, at least in hospital settings where nurses or secretaries often gather such information. Nevertheless, the quality of and the doctors trust in the data regarding all of the SCR’s functions – particularly the pharmaceutical summary and the critical information section – are both of great importance when drawing conclusions about what the SCR represents to doctors. As previously stated, while the pharmaceutical summary gathers information automatically from a national pharmaceutical database called The Prescription Provider on a daily basis, keeping the other functions updated requires continuously manually entering relevant critical information. This means extra record keeping and the duplication of work for doctors both in primary and specialist care settings. Our study indicates that particularly the doctors in busy hospital practices prioritise their local or regional health records over the national SCR. Hence, automatically synchronised information, as in the pharmaceutical summary, increases trustworthiness by removing the responsibility from the doctors and reducing the opportunity for the introduction of errors into patient records.

In sum, our study has found that trustworthiness is a particularly important issue in relation to doctors’ use of and experiences with the SCR in Norway. In the future, when designing and implementing complex technologies with pervasive implications, developers and policy makers must not only consider which information health professional need in clinical practice – they should also consider trustworthiness in relation to these data and data sources. In a future study, we will therefore compare doctors’ use of and experiences with the manually updated data in the SCR with another national tool containing only automatically updated data sources for an in-depth analysis of trust.

## Additional file

Additional file 1: Interview guide. (DOCX 13 kb)

## Abbreviations

EHR: Electronic Health Record
GP: General Practitioner
SCR: Summary Care Record
