# Supplementary material for: The Norwegian National Summary Care Record: a qualitative analysis of doctors’ use of and trust in shared patient information
Source: BMC Health Serv Res. 2018 Apr 6;18:252. doi: 10.1186/s12913-018-3069-y (PMC5889579; doi:10.1186/s12913-018-3069-y)
Supplement: Supplementary file 1 — Interview guide. (DOCX 13 kb) [file 12913_2018_3069_MOESM1_ESM.docx]

Interview guide; “The Norwegian National Summary Care Record. A qualitative analysis of doctors' use of and trust in shared patient information”

The study has an explorative research design where we were interested in the doctors’ personal experiences and stories about the summary care record (SCR). We started all the interviews by informing about the study design and by introducing the two main questions.

1. Can you please tell us about yourself as a doctor?
2. What does the SCR mean to you?

We emphasised that there were no right or wrong answers, and encouraged the doctors to reflect on what the Summary care record meant to them and to talk freely about their opinions. We also said that we were not going to interrupt but would take notes for follow-up questions if we felt this was necessary.
